# DNA Hypermethylation Involves in the Down-Regulation of Chloride Intracellular Channel 4 (CLIC4) Induced by Photodynamic Therapy

**DOI:** 10.3390/biomedicines9080927

**Published:** 2021-07-31

**Authors:** Pei-Chi Chiang, Pei-Tzu Li, Ming-Jen Lee, Chin-Tin Chen

**Affiliations:** 1 Department of Biochemical Science and Technology, College of Life Science, National Taiwan University, Taipei 10617, Taiwan; peggier309@gmail.com (P.-C.C.); peitzuli@gmail.com (P.-T.L.); 2 Department of Neurology and Medical Genetics, National Taiwan University Hospital, Taipei 10012, Taiwan; mjlee@ntu.edu.tw

**Keywords:** CLIC4, DNA methylation, oxidative stress, PDT

## Abstract

The altered expression of chloride intracellular channel 4 (CLIC4) was reported to correlate with tumor progression. Previously, we have shown that the reduced cellular invasion induced by photodynamic therapy (PDT) is associated with suppression of CLIC4 expression in PDT-treated cells. Herein, we attempted to decipher the regulatory mechanisms involved in PDT-mediated CLIC4 suppression in A375 and MDA-MB-231 cells in vitro. We found that PDT can increase the expression and enzymatic activity of DNA methyltransferase 1 (DNMT1). Bisulfite sequencing PCR further revealed that PDT can induce hypermethylation in the *CLIC4* promoter region. Silencing DNMT1 rescues the PDT-induced CLIC4 suppression and inhibits hypermethylation in its promoter. Furthermore, we found tumor suppressor p53 involves in the increased DNMT1 expression of PDT-treated cells. Finally, by comparing CLIC4 expression in lung malignant cells and normal lung fibroblasts, the extent of methylation in *CLIC4* promoter was found to be inversely proportional to its expression. Taken together, our results indicate that CLIC4 suppression induced by PDT is modulated by DNMT1-mediated hypermethylation and depends on the status of p53, which provides a possible mechanistic basis for regulating CLIC4 expression in tumorigenesis.

## 1. Introduction

Photodynamic therapy (PDT) has been developed as an alternative approach for cancer treatment [1]. PDT is based on the administration of exogenous photosensitizer followed by selective light irradiation onto tissue lesion to trigger the production of reactive oxygen species (ROS) [2]. The tumoricidal action of PDT involves the cancer cell killing, disruption of tumor vasculature with the following local inflammation. There are many studies conducted to investigate the molecular mechanisms involved in PDT-mediated cell death [3,4,5]. In addition to cell death, a few other studies reported that the primary tumor treated by PDT exhibits a decreased incidence of distant metastasis [6,7,8]. Our previous findings further revealed that the reduced invasiveness of PDT-treated cells relates to the decreased expression of chloride intracellular channel 4 (CLIC4) [9]. However, the molecular mechanisms involved in regulating the expression of CLIC4 by PDT-induced oxidative stress remain elusive.

The family of chloride intracellular channel (CLIC) proteins are ubiquitously expressed in various tissues and implicated in diverse physiologic functions [10,11]. CLIC4 is the most studied member in the CLIC family and found in soluble and membrane bound forms [12,13]. The biological functions of CLIC4 are found to be involved in regulating cell cycle arrest, apoptosis, metabolic stress, cytoskeletal organization, cell differentiation, and morphogenesis [14]. Knockdown CLIC4 in cultured cells can reduce cell proliferation and migration ability as well as induce apoptosis [14,15]. Expressing antisense CLIC4 in tumors derived from transplanting these cells into nude mice may further demonstrate the role of CLIC4 in tumor progression. Down-regulation of CLIC4 in tumors inhibits tumor growth, increases tumor apoptosis and reduces tumor cell proliferation [14]. In addition, loss of CLIC4 in tumor cells as well as gain in tumor stroma cells have been identified in multiple human cancers, which represents the malignant progression [11,16]. Up-regulation of CLIC4 in tumor stroma enhances the growth of cancer xenografts and significantly involves in the process of the transforming growth factor (TGF)-β-mediated myofibroblast conversion, which is a hallmark of a nurturing tumor microenvironment [17,18,19]. Recently, CLIC4 has been found to have broad prospects as a serum/tissue biomarker and therapeutic target for epithelial ovarian cancer. Compared with normal and benign controls, the level of CLIC4 protein was significantly increased in serum from ovarian cancer patients. Increased CLIC4 expression is considered a negative indicator of patient survival [20]. Although CLIC4 was reported to be implicated in tumorigenesis, no mutation or deletion of the *CLIC4* gene was found in tumor tissues with different stage of pathological condition [11]. Therefore, the molecular mechanisms underlying the regulation of CLIC4 expression are still not clear.

The absence of mutations in the *CLIC4* gene during tumorigenesis implies that epigenetic or post-translational modifications may be involved in its expression control. The GC-rich in the *CLIC4* promoter region suggests that DNA methylation may play a role in mediating CLIC4 transcription. DNA methylation is one category of epigenetics, which has been defined as the study of stable and heritable alterations of chromatin states and dynamics as well as in gene expression potential but do not attributable to mutations in the primary DNA sequence [21,22]. DNA methylation occurs via covalently adding of a methyl group to cytosine residue in CpG dinucleotides, which are concentrated in a short stretch of DNA called CpG island. CpG island is defined as a region with at least 200 bp, the proportion of GC content greater than 50%, and observed to expected CpG ratio (O/E) greater than 0.6 [23]. The CpG islands usually locate in the 5’ end of the candidate genes and nearly 60% of them are in the promoter region [24,25]. Hypomethylation of regulatory sequences, in general, tends to correlate with an increased gene expression, while hypermethylation is typically associated with transcriptional silencing [26]. Growing evidences demonstrate that aberrant promoter methylation of genes can be induced by reactive oxygen species [27,28]. As mentioned, ROS are the major cytotoxic agents responsible for cellular damage induced by PDT [29,30,31]. Although it has been shown that oxidative stress can promote hypermethylation and further suppress gene expression, there are few studies regarding whether PDT-induced oxidative stress may affect DNA methylation. Demyanenko et al. found that 5-aminilevulinic acid (ALA)-mediated PDT alters expression of proteins involved in epigenetic regulation in the mouse cerebral cortex [32]. However, whether there is any change in DNA methylation in PDT-treated cells remains unknown.

In the present study, we investigated the status and the associated molecular mechanisms of DNA methylation in the promoter region of *CLIC4* gene in PDT-treated cells. Our results indicate that suppression of CLIC4 expression by PDT-induced oxidative stress is modulated by DNA methylation in a DNA methyltransferases 1 (DNMT1)-dependent manner and relies on the status of tumor suppressor protein p53. Furthermore, analyzing the methylation status of *CLIC4* promoter in lung malignant cells and normal lung fibroblast cell lines reveal that the extent of methylation status in *CLIC4* promoter is inversely proportional to its expression level. This work provides an insight into a new mechanism by which PDT induces molecular alterations through DNA methylation and suggests a possible regulatory mechanism of CLIC4 during tumorigenesis.

## 2. Materials and Methods

### 2.1. Cell Culture and Photodynamic Treatment

Human melanoma A375 cells and lung adenocarcinoma epithelial A549 cells were cultured in Dulbecco’s modified Eagle’s medium (DMEM) supplemented with 10% (*v/v*) fetal bovine serum (FBS). Human breast adenocarcinoma MDA-MB-231 cells, lung ad-enocarcinoma CL1-0 cells, non-small cell lung cancer H1299 cells and large cell lung cancer H460 cells were cultured in RPMI1640 medium supplemented with 10% (*v/v*) FBS. Human lung fibroblast MRC-5 cells were cultured in Eagle’s minimum essential medium (MEM) supplemented with 10% (*v/v*) FBS. All cells were grown at 37 °C under 5% CO_2_. MRC-5 was obtained from American Type Culture Collection (ATCC, Manassas, VA, USA). A375, MDA-MB-231, A549, H1299, H460 and PC3 were obtained from The National Health Research Institutes (NHRI) Cell Bank (Taipei, Taiwan). Both cell lines of A375 and MDA-MB-231 were authenticated using the PromegaGenePrint 10 System (Promega, Madison, WI, USA) and analyzed by ABI PRISM 3730 GENETIC ANALYZER and GeneMapper software V3.7 (Applied Biosys-tems, Carlsbad, CA, USA). CL1-0 was a kind gift from Dr. Pan-Chyr Yang’s Lab. (Department of Internal Medicine, National Taiwan University Hospital, Taipei, Taiwan) [33]. For photodynamic treatment, cells were incubated with 1 mM ALA (Sigma-Aldrich, St. Louis, MO, USA) in serum free medium for 3 h and then exposed to specific dose of light as indicated that corresponds to LD_50_. Light source for ALA-PDT is a home-made high-power LED array with the wavelength centered at 635 ± 5 nm. Immediately after light irradiation, cells were cultured in complete medium until further analysis.

### 2.2. Western Blotting

Immunoblot analysis was carried out as described previously [34]. Briefly, cell lysates were separated by SDS-PAGE gel. Protein samples were transferred to a nitrocellulose membrane, blocked with 5% (*w/v*) skim milk, and probed with specific primary antibodies. The primary antibodies used are anti-CLIC4 antibody (Abcam, Cambridge, UK), anti-p53 antibody (Cell Signaling Technology, Beverly, CA, USA) and anti-DNMT1 antibody (Epitomics, Burlingame, CA, USA). The horseradish peroxidase (HRP)-conjugated secondary antibody was used and the immunocomplex was visualized by Chemiluminescence Reagent Plus (Blossom Biotechnology Inc., Boston, MA, USA). Chemiluminescence detection was measured directly by a Biospectrum 810 Imaging System (UVP, Upland, CA, USA). For the loading control, the membranes were further stripped and re-probed with anti-GAPDH antibody (GeneTex, Irvine, CA, USA) and anti-Tubulin antibody (Cell signaling, Boston, MA, USA). The origin immunoblots and the replicated data in this study are shown in Supplementary Figure S7 online. Band blots for each protein were from the same set of samples. Before hybridizing with each antibody, according to the expected molecular weight of target proteins, blots were cropped from different sections of the same whole blot to conserve reagents and avoid signal fading caused by striping and re-probing. However, we did not retain the full-length photograph of the blot when experiments were performed.

### 2.3. Reverse Transcription-PCR Analysis

To assess gene expression, total RNA from cells was extracted by using Trizol reagent (Invitrogen, Carlsbad, CA, USA) according to the manufacturer’s instructions. The obtained RNA was reverse transcribed with SuperScript II reverse transcriptase (Invitrogen, Carlsbad, CA, USA) based on the manufacturer’s protocol. The primer sequences used for PCR are as follows: CLIC4, 5′-gCAgTgATggTgAAAgCATAg-3′ (forward) and 5′-TATAAATggTgggTgggTCC-3′ (reverse); DNMT1, 5′-ACCgCTTCTACTTCCTCgAggCCTA-3′ (forward) and 5′-gTTgCAgTCCTCTgTgAACACTgTgg-3′ (reverse); p53, 5′-TTggATCCATgTTTTgCCAACTggCC-3′ (forward) and 5′-TTgAATTCAggCTCCCCTTT-CTTgCg-3′ (reverse); β-actin, 5′-TggACTTCgAgCAAgAgATgg-3′ (forward) and 5′-ATCTCCTTCTgCATCCTgTCg-3′ (reverse); GAPDH, 5′-gACCACAgTCCATgCCATCA-3′ (forward) and 5′-gTCCACCACCCTgTTgCTgTA-3′ (reverse). RT-PCR was performed as previously described [34]. The PCR products were resolved on a 2% (*w/v*) non-denaturing agarose gel and analyzed using EtBr staining. The original images of electrophoresis gels in this study are shown in Supplementary Figure S8 online. The band intensities of PCR products were measured by analyzing the gel images on the ImageJ software. The mRNA expression level of each gene was normalized to *β-actin* and *GAPDH* for RT-PCR and real-time PCR, respectively. To assess mRNA expression, cDNA product was also used as a template for real-time PCR analysis using ABI Fast SYBR Green Master Mix Kit (Thermo Fisher Scientific, Waltham, MA, USA) with ABI StepOne system (Thermo Fisher Scientific, Waltham, MA, USA). Results were expressed as fold change over the controls.

### 2.4. RNA Interference

shRNA vectors were obtained from the National RNAi Core Facility (Academia Sinica, Taipei, Taiwan). The target sequence for DNMT1 and p53 shRNAs were as follows: shDNMT1#1, 5′-CCgggCCCAATgAgACTgACATCAACTCgAgTTgATgTCATCTCATTgggCTTTTT-3′; shDNMT1#2, 5′-CCggCgACTACATCAAAggCAgCAACTCgAgTTgCTgCCTTTgATgTAgTCgTTTTT-3′; shp53#1, 5′-CCggCACCATCCACTACAACTACATCTCgAgATgTAgTTgTAgTggATggTgTTTTT-3′; shp53#2, 5′-CCggCggCgCACAgAggAAgAgAATCTCgAgATTCTCTTCCTCTgTgCgCCgTTTTT-3′; shp53#3, 5′-CCgggAgggATgTTTgggAgATgTACTCgAgTACATCTCCCAAACATCCCTCTTTTT-3′. Templates were inserted into lentiviral plasmids (pLKO.1-puro). Cells were transiently transfected with a validated empty vector, DNMT1 or p53 shRNA using Lipofectamine 2000 (Invitrogen, Carlsbad, CA, USA). After six hours of transfection, the cells were replaced with fresh medium and incubated at 37 °C for another 72 h. On the following day, the treated cells were harvested and used for further analysis.

### 2.5. DNA Methyltransferases Activity

The sampled cells were harvested, and the nuclear protein samples were prepared with the nuclear extraction reagent (Panomics Inc., Fremont, CA, USA). The DNMT enzymatic activity assay was measured using DNMT Activity/Inhibition Assay Kit (Active Motif Inc., Carlsbad, CA, USA) according to the manufacturer’s instruction. All the assays were performed in duplicate in three sets of independent experiments. Background levels were determined in assays in which the template DNA was excluded.

### 2.6. Bisulfite Genomic Sequencing

Methylation status of the CLIC4 promoter was assessed by bisulfite sequencing. Genomic DNA was extracted from the sampled cells by the Quick-gDNA MicroPrep (Zymo Research Inc., Irvine, CA, USA) and subjected to bisulfite conversion using EZ DNA Methylation Kit (Zymo Research Inc., Irvine, CA, USA) according to the manufacturer’s recommendations. For each conversion, 0.5 μg of genomic DNA was used. PCR was performed for amplifying the promoter CpG island from the *CLIC4* gene. The primer sequences are as follows: BSP #1 (forward), 5′-TTTTTTAgAggATTTgggAAAT-3′; BSP #1 (reverse), 5′-CTTAACAACCAACATATTCACAAA-3′; BSP #2 (forward), 5′-TAgTTATTTgggAggTTgAgg-3′; BSP #2 (reverse), 5′-ACRCCCCACAACTAATAAA-3′; BSP #3A (forward), 5′-TgAgTTTTggggTgTTg-3′; BSP #3A (reverse), 5′-AAAAAATTCCCCAAAAACC-3′; BSP #3B (forward), 5′-TgTTAggTTYgggTTTTT-3′; BSP #3B (reverse), 5′-AACCRAAAAAAAAACCTCT-3′; BSP #3C (forward), 5′-gggYgTYgTAgAggTT-3′; BSP #3C (reverse), 5′-CRCCRAAAACRAAAC-3′; BSP #3D (forward), 5′-gAgAgTTTYgAggYgT-3′; BSP #3D (reverse), 5′-ACCACRACTTCAACTCCT-3′; BSP #4 (forward), 5′-gTgTTgAggAgTTgAAgTYgT-3′; BSP #4 (reverse), 5′-TACCCAAAACAAAAAAACACAA-3′. The amplified fragments were subcloned into the pCR4-TOPO vector by TA cloning (Invitrogen, Carlsbad, CA, USA) and the colony PCR products were submitted for nucleotide sequencing.

### 2.7. Statistical Analysis

All experiments were performed and repeated at least three times. Data in bar graphs are expressed as the mean and standard deviation from 3 independent experiments. The statistical significance of experimental data was evaluated by two-tailed Student’s *t*-test and considered statistically significant with * *p* < 0.05, ** *p* < 0.01 and *** *p* < 0.001.

## 3. Results

### 3.1. Inhibition of DNA Methylation Restores PDT-Induced Reduction of CLIC4 Expression

**Figure 1:**
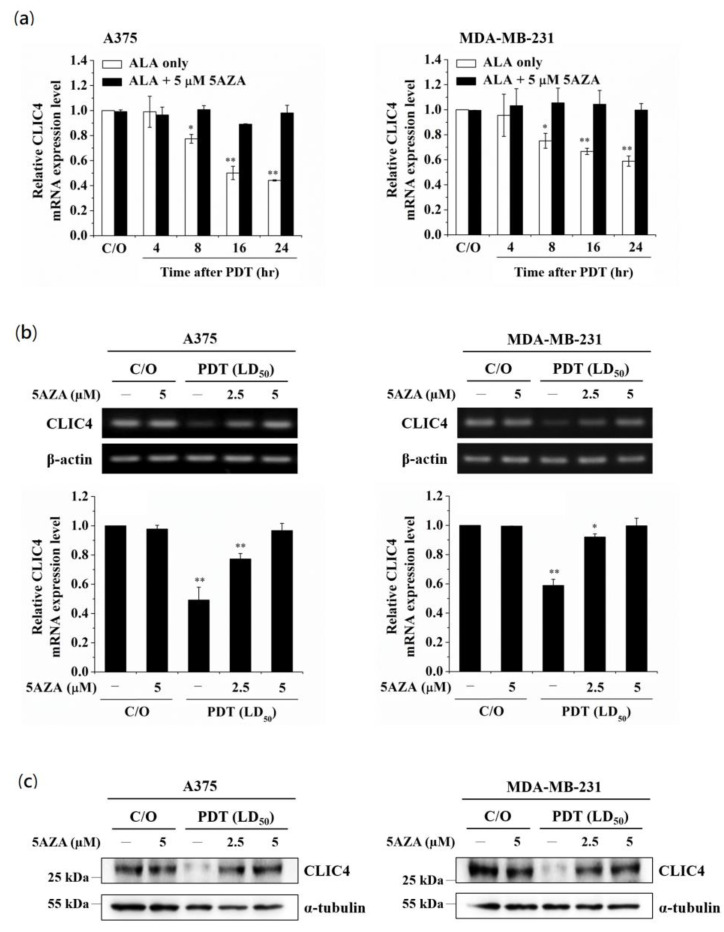
Inhibition of DNA methylation relieves the suppression of CLIC4 expression induced by PDT-mediated oxidative stress. Cells were pre-incubated with 5AZA for 2 h prior to light irradiation. For ALA-PDT, cells were pre-incubated with 1 mM ALA for 3 h and then exposed to specific wavelength (635 ± 5 nm) of light, 2 J/cm^2^ and 4 J/cm^2^ for A375 (left panel) and MDA-MB-231 (right panel) cells, respectively. To verify the mRNA expression level of CLIC4, total RNA samples were isolated (**a**) from PDT-treated cells at the time indicated (4, 8, 16 and 24 h after ALA-PDT) with or without pre-incubation of 5 µM 5AZA, and (**b**) from PDT-treated cells at 24 h as indicated with or without pre-incubation of 2.5 and 5 µM 5AZA. The relative mRNA expression level of each gene was measured by RT-PCR and normalized to β-actin; (**c**) The protein expression level of CLIC4 was analyzed by Western blotting, and α-tubulin was used as an internal control. The control group was cells only treated with ALA w/o light irradiation. For electrophoretic gels, the same volume of each PCR product was loaded, and band blots for each gene were cropped from different gels. For immunoblots, equal volume of each sample with the same concentration was loaded into each well, and the blots were cropped from different sections of the same gel. Data represent from three independent experiments. Each bar shown is the mean fold change relative to control ± SD. Results are considered to be statistically significant at * *p* < 0.05 and ** *p* < 0.01.

Previously, we have shown that PDT can induce chromatin modification by regulating the expression and activity of histone acetyltransferase p300 (p300HAT) [31], suggesting epigenetic modifications may play an important role in PDT-mediated gene regulation. To examine whether epigenetic modifications involve in regulating PDT-induced suppression of CLIC4 expression, we first used a DNMT inhibitor and a histone deacetylase (HDAC) inhibitor, 5-azacytidine (5AZA) and Trichostatin A (TSA), to block DNA methylation and histone acetylation before PDT treatment. As shown in Figure 1a, 5AZA can significantly restore the mRNA expression level of CLIC4 in PDT-treated A375 and MDA-MB-231 cells. The effect of 5AZA was shown in a dose-dependent manner in reversing the decreased levels of CLIC4 mRNA (Figure 1b) and protein (Figure 1c) expression in PDT-treated cells. Pre-treatment of 5AZA did not affect the viability of PDT-treated cells (see Supplementary Figure S1 online), indicating that CLIC4 expression restored by 5AZA treatment was not accompanied by a change in cell viability. Meanwhile, we also found that the mRNA expression level of CLIC4 after PDT was not affected by TSA (see Supplementary Figure S2 online). These results indicate that the regulatory mechanisms involved in the suppressed expression of CLIC4 may relate to the DNA methylation in PDT-treated cells.

### 3.2. Oxidative Stress Mediated by PDT Increases the Expression and Activity of DNMT1

**Figure 2:**
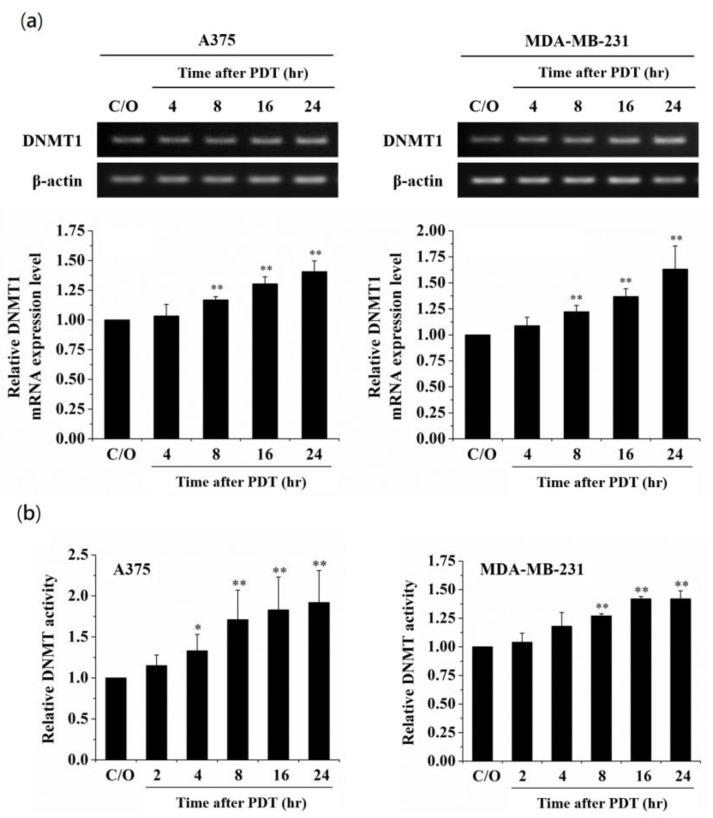
Increase of mRNA expression level and enzymatic activity of DNMT1 in PDT-treated cancer cells. (**a**) RT-PCR was performed to analyze the mRNA expression level of DNMT1 in A375 (left panel) and MDA-MB-231 (right panel) cells at the time indicated after ALA-PDT. The light dose for A375 and MDA-MB-231 cells is 2 J/cm^2^ and 4 J/cm^2^, respectively. The relative mRNA expression level of each gene was measured by RT-PCR and normalized to β-actin. The same volume of each PCR product was loaded, and band blots for each gene were cropped from different gels; (**b**) Nuclear protein samples were extracted from PDT-treated cells. 5 µg protein of each sample was used for analyzing the DNMT activity assay. The control group was cells only treated with ALA w/o light irradiation. Data represent from three independent experiments. Each bar shown is the mean fold change relative to control ± SD. Results are considered to be statistically significant at * *p* < 0.05 and ** *p* < 0.01.

**Figure 3:**
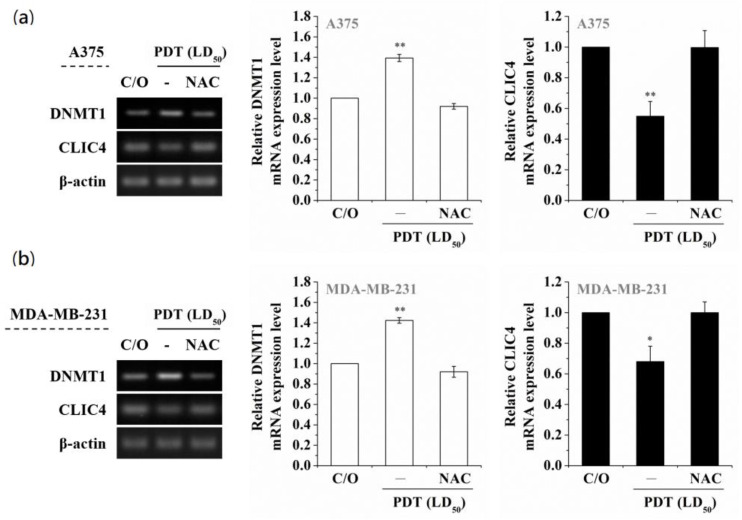
Scavenging ROS production suppresses the PDT-induced increase of DNMT1 and constrains the following CLIC4 suppression. Cells were co-incubated with both 1 mM ALA and 5 mM NAC for 3 h and then exposed to specific wavelength (635 ± 5 nm) of light, 2 J/cm^2^ and 4 J/cm^2^ for (**a**) A375 and (**b**) MDA-MB-231 cells, respectively. Total RNA samples were isolated from PDT-treated cells 24 h after ALA-PDT to analyze the mRNA expression level. The relative mRNA expression level of each gene was measured by RT-PCR and normalized to β-actin. The control group was cells only treated with ALA w/o light irradiation. The same volume of each PCR product was loaded, and band blots for each gene were cropped from different gels. Data represent from three independent experiments. Each bar shown is the mean fold change relative to control ± SD. Results are considered to be statistically significant at * *p* < 0.05 and ** *p* < 0.01.

Among the DNMT protein family, DNMT1 is the most abundant one in mammalian cells, which plays a role in methylating newly replicated DNA [35]. In addition, alterations of DNMT1 induced by oxidative stress have been observed in hydrogen peroxide-treated cells [36,37]. To examine whether PDT-mediated alteration of DNA methylation is due to the activation of DNMT1, we first analyzed the DNMT1 mRNA expression level in PDT-treated A375 and MDA-MB-231 cells. As shown in Figure 2a, the DNMT1 mRNA expression level was significantly elevated in a time-dependent manner following PDT, which was related to the increase of DNMT1 enzymatic activity (Figure 2b). Moreover, pre-treatment of A375 and MDA-MB-231 cells with the ROS scavenger, N-acetyl cysteine (NAC), could reverse the PDT-induced up-regulation of DNMT1 and suppression of CLIC4 expression (Figure 3). These results indicate that PDT-induced oxidative stress can up-regulate the expression and activity of DNMT1, which may further down-regulate the CLIC4 expression via DNA methylation.

### 3.3. DNMT1 Is Required to Down-Regulate CLIC4 Expression in PDT-Treated Cells

**Figure 4:**
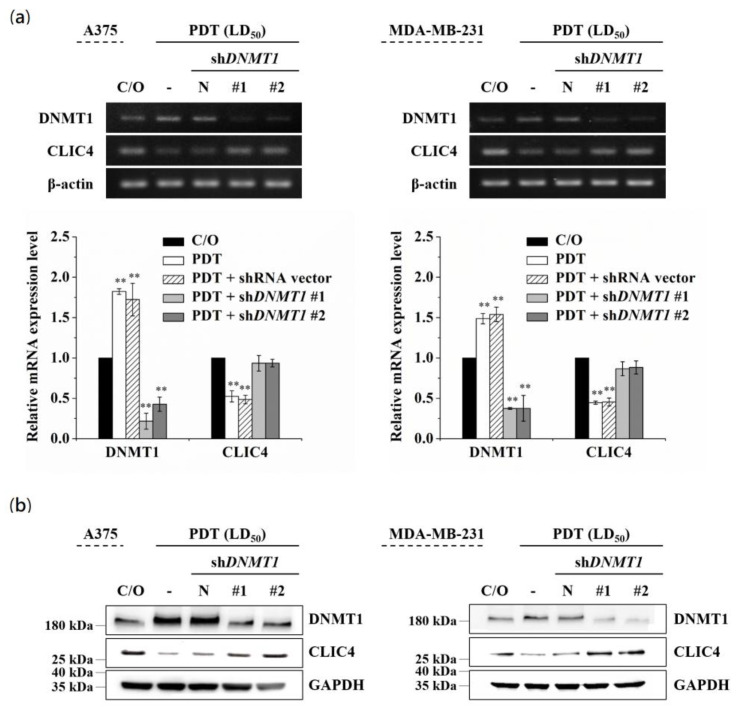
Down-regulation of DNMT1 restored the suppressed expression of CLIC4 in PDT-treated cells. A375 (left panel) and MDA-MB-231 (right panel) cells were transfected with either the shDNMT1 (denoted as shDNMT1 #1 and #2) or an empty shRNA vector (pLKO.1, as negative control). (**a**) Total mRNA and (**b**) protein samples were isolated from cells 24 h after ALA-PDT. The light dose for A375 and MDA-MB-231 cells is 2 J/cm^2^ and 4 J/cm^2^, respectively. The relative mRNA expression level of each gene was measured by RT-PCR and normalized to β-actin. The protein expression levels of DNMT1 and CLIC4 were analyzed by Western blotting, and GAPDH was used as an internal control. The control group was cells only treated with ALA w/o light irradiation. For electrophoretic gels, the same volume of each PCR product was loaded, and band blots for each gene were cropped from different gels. For immunoblots, equal volume of each sample with the same concentration was loaded into each well, and the blots were cropped from different sections of the same gel. Data represent from three independent experiments. Each bar shown is the mean fold change relative to control ± SD. Results are considered to be statistically significant at ** *p* < 0.01.

To further verify whether the increased DNMT1 is required for down-regulating CLIC4 in PDT-treated cells, we used two different DNMT1-specific short hairpin RNAs (shRNAs) to knockdown the endogenous DNMT1 followed by assessing the expression of CLIC4. As shown in Figure 4, both shDNMT1 #1 and #2 constructs could effectively reduce the mRNA expression level of DNMT1 in A375 and MDA-MB-231 cells (Figure 4a). The same results were observed in protein expression level analyzed by immunoblotting (Figure 4b). PDT-induced down-regulation of CLIC4 was significantly abrogated in A375 and MDA-MB-231 cells transiently transfected with shDNMT1 but not in cells transfected with the backbone shRNA vector, which indicates a reciprocal change between DNMT1 and CLIC4 expression in the knockdown cells post PDT treatment. These results suggest that DNMT1 is required in PDT-induced CLIC4 suppression.

### 3.4. The Increased DNMT1 Relates to the Activated p53 in PDT-Treated Cells

**Figure 5:**
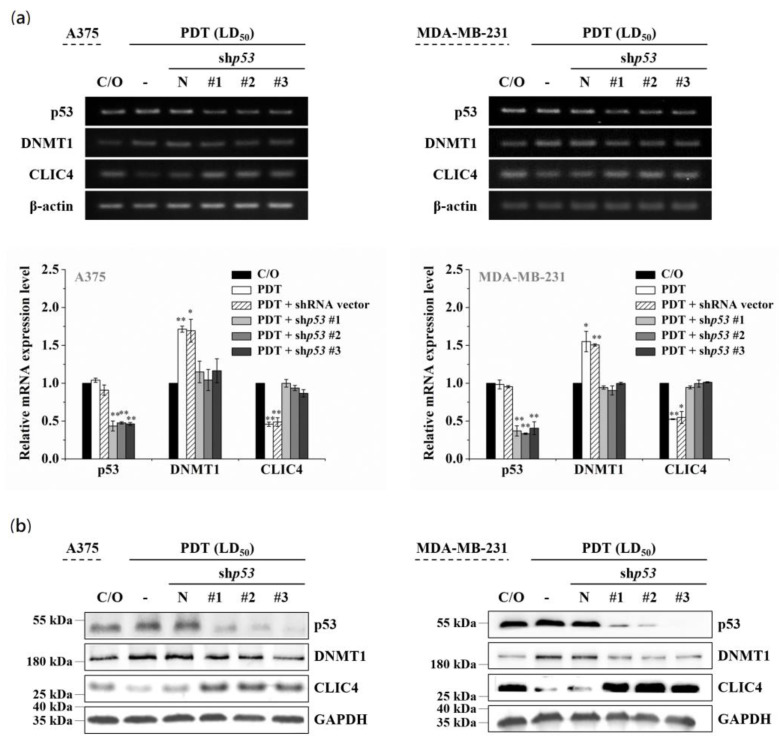
p53 involves in the altered expression of DNMT1 and CLIC4 in PDT-treated cells. A375 (left panel) and MDA-MB-231 (right panel) cells were transfected with either the shp53 (denoted as #1, #2 and #3) or an empty shRNA vector (pLKO.1 as negative control). (**a**) Total mRNA and (**b**) protein samples were isolated from cells 24 h after ALA-PDT. The light dose for A375 and MDA-MB-231 cells is 2 J/cm^2^ and 4 J/cm^2^, respectively. The relative mRNA expression level of each gene was measured by RT-PCR and normalized to β-actin. The control group was cells only treated with ALA w/o light irradiation. For electrophoretic gels, the same volume of each PCR product was loaded, and band blots for each gene were cropped from different gels. For immunoblots, equal volume of each sample with the same concentration was loaded into each well, and the blots were cropped from different sections of the same gel. Data represent from three independent experiments. Each bar shown is the mean fold change relative to control ± SD. Results are considered to be statistically significant at * *p* < 0.05 and ** *p* < 0.01.

It has been shown that DNMT1-mediated methylation is stimulated by the activation of p53 protein [38,39]. On the other hand, PDT-induced up-regulation of p53 has been found in various PDT-treated cells [40,41]. Compared to the cells either untreated or only incubated with ALA, the level of p53 protein indeed was considerably increased following PDT (see Figure S3 online). Therefore, we further addressed whether the DNMT1 up-regulation is associated with the activation of p53 in PDT-treated cells. As shown in Figure 5a,b, the mRNA and protein expression levels of p53 were significantly reduced in three different p53-specific shRNAs transfected cells, which also shows a reciprocal change in mRNA and protein levels between DNMT1 and CLIC4 after PDT. However, knockdown of p53 in cells without PDT treatment would not affect the expression of DNMT1 or CLIC4 (see Figure S4 online). These findings indicate that p53 plays an important role in modulating the increased DNMT1 expression in PDT-treated cells, which may further mediate the suppression of CLIC4 expression by promoting DNA methylation.

### 3.5. PDT Induces Suppression of CLIC4 Expression by Methylating its Promoter Region in A DNMT1-Dependent Manner

**Figure 6:**
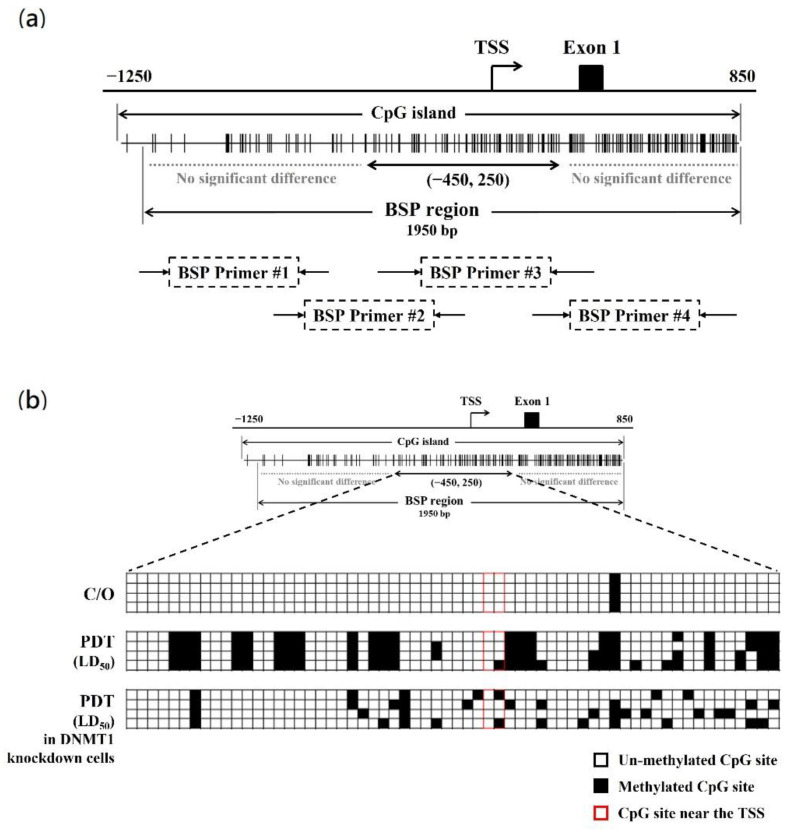
PDT induces the methylation alteration of CLIC4 promoter region in a DNMT1-dependent manner. (**a**) Schematic diagram of the CpG island of CLIC4 promoter. The distribution of CpGs crosses over the promoter and the first exon of CLIC4 gene. The transcriptional start site (TSS) is indicated. Each vertical bar represents the presence of a CpG dinucleotide. BSP1, BSP2, BSP3 and BSP4 represent the regions selected for bisulfite sequencing PCR (marked as BSP region); (**b**) Direct sequencing of bisulfite PCR products was performed in melanoma A375 cells: no-treatment control group, PDT-treated cells, and PDT-treated DNMT1-knockdown cells. Genomic DNA was extracted from cells 24 h after ALA-PDT with the light dose of 2 J/cm^2^. Quantification of the bisulfite-sequencing data, shown as the percentage of DNA methylation (*n* = 4) of the region among the CLIC4 gene from position nt. −450 to +250. Methylation status of cytosine is shown as follows: filled square (◼), methylated; open square (◻), unmethylated.

To further explore whether PDT-induced suppression of CLIC4 expression correlates to the DNA hypermethylation, the DNA methylation status of *CLIC4* promoter region in PDT-treated cells was examined. The methylation of CpG islands in the promoter region (−1214 to +795; the transcription start site (TSS) at +1) was assessed by bisulfite sequencing PCR (BSP). Specific primers were used to amplify the region spanning from nucleotide position −1126 to +973, which encompasses 181 CpG sites (Figure 6a). As shown in Figure 6b, compared to the control group, the methylation level at the position between −450 and +250 (containing 62 CpG sites) of *CLIC4* gene showed a substantial increase in PDT-treated cells. The percentage of methylated CpG islands in the assay area is 38.3% for the PDT-treated but only 1.6% for the untreated cells. These results indicate that PDT induces DNA methylation in the promoter region of *CLIC4* may contribute to its suppressed expression. To further investigate whether such a methylation is attributed to the DNMT1 function, the methylation status of *CLIC4* promoter region in A375 cells transfected with DNMT1-specific shRNA was also evaluated. After PDT treatment, there is 15.3% of methylation at the candidate region (nt. −450 to +250) in DNMT1 knockdown cells. These findings suggest that hypermethylation in the *CLIC4* promoter region induced by PDT is mainly DNMT1-dependent.

### 3.6. CLIC4 Expression Is Regulated by DNA Hypermethylation in Its Promoter Region

**Figure 7:**
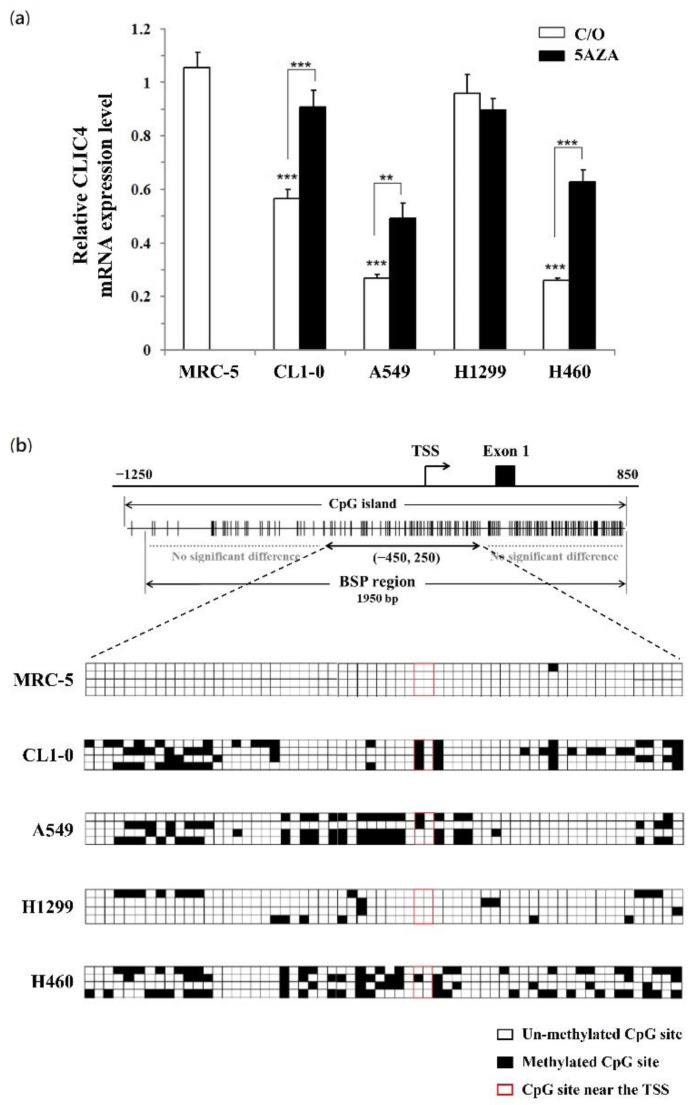
The methylation status of CLIC4 promoter correlates with its mRNA levels in different cell lines. (**a**) The real-time PCR was performed to analyze the mRNA expression level of CLIC4 in normal lung fibroblast cells, MRC-5; and lung cancer cells, CL1-0, A549, H1299, H460. Cells were treated with or without 5 µM 5AZA for 2 h. To compare the mRNA expression level of CLIC4 in different cell line, MRC-5 cells w/o 5AZA treatment were used as a control group. The relative mRNA expression level of each gene was normalized to GAPDH. Data represent from three independent experiments. Each bar shown is the mean fold change relative to control ± SD. Results are considered to be statistically significant at ** *p* < 0.01 and *** *p* < 0.001; (**b**) Direct sequencing of bisulfite PCR products was performed to explore the methylation status of CLIC4 promoter region. Quantification of the bisulfite-sequencing data, shown as the percentage of DNA methylation (*n* = 4) of the region among the *CLIC4* gene from position nt. −450 to +250. Methylation status of cytosine is shown as follows: filled square (◼), methylated; open square (◻), unmethylated.

The expression of CLIC4 is commonly reduced in many human cancers, including skin, breast, prostate, and lung tumors [11,42]. However, no evidence showed a mutation or deletion in the CLIC4 gene during tumorigenesis. As PDT-mediated CLIC4 suppression is correlated to the induction of methylation in its promoter region, DNA methylation status of the promoter region might contribute to the regulation of CLIC4 expression in cancer cells. In this regard, we first studied the relative mRNA expression level of CLIC4 in different lung cancer cell lines and normal lung fibroblast cell line, MRC-5. As shown in Figure 7a, the mRNA expression level of CLIC4 in normal MRC-5 fibroblasts was significantly higher than the malignant CL1-0, A549 and H460 cancer cells, but similar to that of H1299 cancer cells. Meanwhile, treatment with DNMT inhibitor, 5AZA, resulted in increased level of CLIC4 mRNA expression in CL1-0, A549 and H460 cancer cells (Figure 7a). The bisulfite sequencing analysis revealed that the CpG sites (nt. −450 to +250) are barely methylated and no methylation around the TSS in MRC-5 and H1299 cells, which express the higher level of CLIC4 mRNA (Figure 7b). In contrast, a considerably higher methylation level in the promoter region, especially heavy methylated around the TSS, was found in CL1-0, A549 and H460 cells with lower CLIC4 mRNA expression level. These results suggest that DNA methylation in the promoter region of CLIC4 might relate to the decreased CLIC4 expression during carcinogenesis.

## 4. Discussion

Our previous study demonstrated that the down-regulation of CLIC4 related to the reduced invasiveness of PDT-treated cells [9]. In this study, we further investigated the regulatory mechanisms involved in PDT-mediated CLIC4 suppression. DNA methylation and histone acetylation are important epigenetic mechanisms involved in transcriptionally regulating gene expression. We have previously demonstrated that the oxidative stress mediated by PDT can significantly up-regulate the activity and expression of p300HAT, which leads to the increased expression of pro-survival molecules such as cyclooxygenase-2 and survivin [31]. However, in this study, we found that the reduced CLIC4 after PDT was not affected by HDAC inhibitor, which indicated that the reduced CLIC4 expression does not relate to the histone acetylation. Demyanenko et al. reported that ALA-PDT can induce the expression of DNA methylation-dependent protein, Kaiso, in the mouse cerebral cortex [32]. Meanwhile, it has been shown that 5AZA can rescue the necrosis of crayfish glial cells induced by photosensitization, indicating DNA methylation may involve in molecular process of PDT-induced cell damage [43]. In this study, we found that the reduced expression of CLIC4 could be reverted in the presence of 5AZA in PDT-treated cells (Figure 1). It has been shown that CLIC4 knockdown can reduce cell proliferation and migration ability as well as induce apoptosis in cultured cells [14,20]. However, we did not find significant difference in the viability of cells pretreated with or without 5AZA after PDT (see Figure S1 online), suggesting the reduced expression of CLIC4 involved in impairing cell migration rather than cell proliferation. In addition, we found that PDT-mediated oxidative stress can up-regulate the expression of DNMT1, and further leads to the suppression of CLIC4 expression through elevating hypermethylation in its promoter region. Altogether, these studies indicate that epigenetic modifications are involved in the regulatory mechanism of PDT-mediated biological consequences, suggesting a treatment modality of combining epigenetic drugs to improve the therapeutic efficacy of photodynamic therapy.

During tumorigenesis, it has been shown that the expression of CLIC4 was down-regulated in tumor cells but up-regulated in stromal cells [11,16]. In addition, up-regulation of CLIC4 involves in the TGF-β–mediated myofibroblast conversion, suggesting its role in malignant progression [18,19]. Previously, we have shown that the reduced invasiveness of PDT-treated cells relates to the decreased CLIC4 expression [9]. In this study, we further found that PDT can up-regulate the expression of DNMT1, leading to the suppression of CLIC4 expression. In this regard, we speculate that the clinical advantages of PDT might not only exert its therapeutic effects at tumor tissues but also reduce the CLIC4 expression in surviving tumor cells and stromal cells to suppress the malignant progression.

*p53,* a tumor suppressor gene, is frequently inactivated in many cancers. p53 protein plays a decisive role of cell fate upon stress stimuli. With different cell lines and photosensitizers, growing evidences demonstrate that p53 activation contributes to cell killing upon PDT [44,45,46,47]. Here, we reported that up-regulation of p53 following PDT relates to the increased DNMT1 expression, leading to hypermethylation of the *CLIC4* promoter region. This epigenetic modification resulted in the decrease of CLIC4 expression in PDT-treated cells. Although p53 is considered as the upstream activator of CLIC4, restoring expression of p53 does not increase CLIC4 expression in cutaneous squamous cell lines [42]. Correspondingly, we also found that silencing of p53 expression in untreated cancer cells would not affect DNMT1 and CLIC4 expression (see Figure S4 online). To clarify the relationship between p53 and CLIC4 under PDT-mediated oxidative stress, we used the following cancer cell lines for further analysis: A375 (wild-type p53), MDA-MB-231 (stabilized mutant p53), and PC3 (p53-null) cancer cell lines [48]. We found that up-regulation of p53 following PDT relates to the increased DNMT1 expression, leading to hypermethylation of the CLIC4 promoter region in A375 cells with wild-type p53. In contrast, PDT did not significantly increase the p53 expression in MDA-MB-231 cell line which has an endogenously high level of the mutant p53. But knockdown of p53 in PDT-treated MDA-MB-231 cells can still prevent the up-regulation of DNMT1 and restore the down-regulation of CLIC4 (Figure 5). For p53-null PC3 prostate cancer cells, PDT can still induce the expression of DNMT1 with further decrease in CLIC4 expression (see Figure S5 online). Therefore, the expression level of p53 might not be the only factor for the expression of DNMT1 and CLIC4 in response to PDT-mediated oxidative stress. In fact, there are two other protein members of the p53 family, p63 and p73, existed in the p53-null PC3 cells [44]. We speculated that in addition to p53, the p53 isoforms might also conduce to the DNMT1-dependent CLIC4 regulation. In fact, as shown in Figure S6 online, PDT significantly increased the mRNA expression levels of p73 and *DNMT1* in the p53-null PC3 cells. Altogether, these results indicate that the p53 family may play an important role in modulating the increased expression of DNMT1 and the following suppressed expression of CLIC4 by promoting DNA methylation in PDT-treated cells. It is noteworthy that PDT did not significantly increase the p53 expression in MDA-MB-231 cell line which has a high level of the mutant p53 (unpublished data). However, the silencing of p53 expression in PDT-treated MDA-MB-231 cells can still prevent the up-regulation of DNMT1 and restore the down-regulation of CLIC4 (Figure 5). These findings suggest that, in addition to p53, other regulatory mechanisms may also be involved in regulating DNMT1-mediated CLIC4 expression in PDT responses.

Considerable evidences implicate that the distribution of CLIC4 expression in many human neoplasms directly correlates with cancer pathogenesis [49,50]. The Yuspa et al. demonstrated that the suppression of CLIC4 expression is not resulting from the gene mutations and suggested other changes such as epigenetic modification may be responsible for the regulation of CLIC4 [11,20]. The promoter region of *CLIC4* shows a high frequency of CpG sites, suggesting the possibility of promoter silencing by methylation. In this study, we employed the direct bisulfite sequencing PCR to examine the methylation status and demonstrated that PDT-induced oxidative stress can suppress the CLIC4 expression by methylating in its promoter, particularly at the region spanning from position nt. −450 to 250. Furthermore, comparing among the different lung cancer cells and the normal fibroblasts, the DNA methylation status of this region was found to be correlated to the mRNA expression level of *CLIC4* (Figure 7). These findings imply that DNA methylation of the promoter region may be a regulatory mechanism of CLIC4 expression in malignant tumor progression.

In summary, this study demonstrated that the down-regulation of CLIC4 after PDT is mediated by the hypermethylation of its promoter region. The regulatory mechanism involves in the increased expression of p53 and following DNMT1, which further induce hypermethylation in the promoter region of *CLIC4*. Exploration of the methylation status of *CLIC4* promoter and its transcribed mRNA level in malignant lung cancer and normal fibroblast cells revealed the highly methylated status in the malignant cells expressing lower mRNA levels of *CLIC4*. In the future, it is worthwhile to further address whether modulating the DNMT1 activity to regulate the CLIC4 expression could become a novel approach to inhibit the tumor malignancy.

## Data Availability

The datasets generated during and/or analyzed during the current study are available from the corresponding author on reasonable request.

## Supplementary Materials

The following are available online at https://www.mdpi.com/article/10.3390/biomedicines9080927/s1, Figure S1: Cell viability in PDT-treated cells with 5-azacytidine (5AZA) pre-treatment. Figure S2: Inhibition of histone deacetylase (HDAC) activity by Trichostatin A (TSA) cannot suppress the PDT-induced reduction of CLIC4, Figure S3: p53 activation following PDT in A375 cells, Figure S4: *DNMT1* and *CLIC4* mRNA expression levels in p53-knockdown A375 cells, Figure S5: PDT induced DNMT1 expression and further suppressed CLIC4 expression in p53-null PC3 prostate cancer cells, Figure S6: PDT increased *DNMT1* and *p73* expression in p53-null PC3 prostate cancer cells, Figure S7: Original scans of immuno-blots used in main text and supplementary figures, Figure S8: Original scans of electrophoresis gel used in main text and supplementary figures.
