# Supplementary material for: DNA Hypermethylation Involves in the Down-Regulation of Chloride Intracellular Channel 4 (CLIC4) Induced by Photodynamic Therapy
Source: Biomedicines. 2021 Jul 31;9(8):927. doi: 10.3390/biomedicines9080927 (PMC8394338; doi:10.3390/biomedicines9080927)
Supplement: Supplementary file 1 [file biomedicines-09-00927-s001.zip › biomedicines-1318354-supplementary.pdf]

Supplementary Information

DNA Hypermethylation Involves in the Down-regulation of Chloride Intracellular Channel 4 (CLIC4) Induced by Photodynamic Therapy

Pei-Chi Chiang [#], Pei-Tzu Li [#], Ming-Jen Lee and Chin-Tin Chen *

[#] The first two authors contributed equally to this work.

* Correspondence: chintin@ntu.edu.tw (CT Chen)

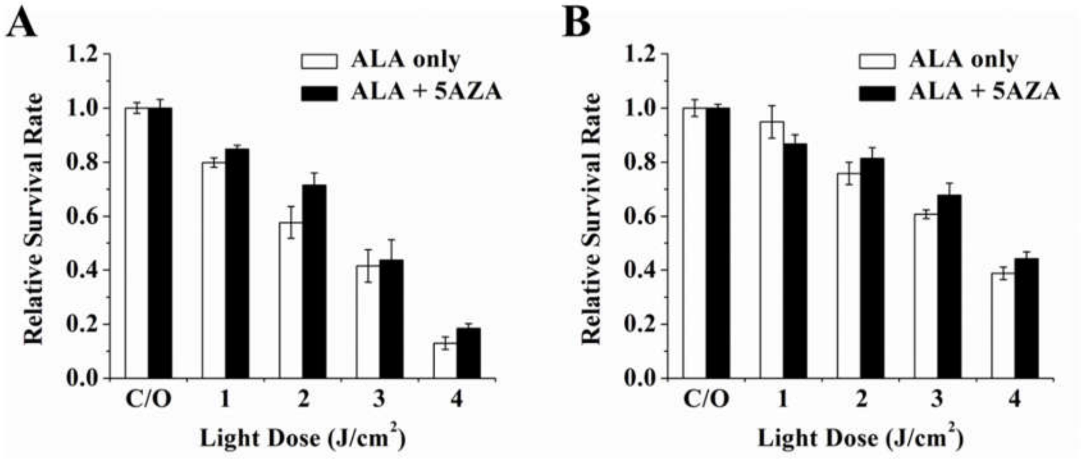

Figure S1. Cell viability in PDT-treated cells with 5-azacytidine (5AZA) pre-treatment. **(A)** A375 and **(B)** MDA-MB-231 cells were pre-incubated with 5 μM 5AZA for 2 hours prior to light irradiation. After ALA-PDT, cell viability was determined by MTT assay 24 hours after light irradiation. Cells receive no PDT treatment were used as a control (C/O). Comparing the groups with only ALA and ALA+5AZA, there was no significant difference between the cell viability after light irradiation. Each bar shown is the mean fold change relative to control ± SD (N=3).

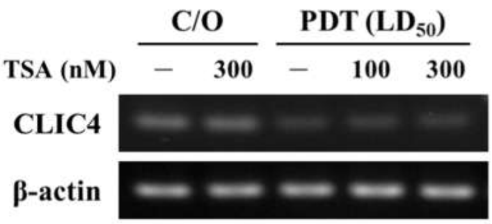

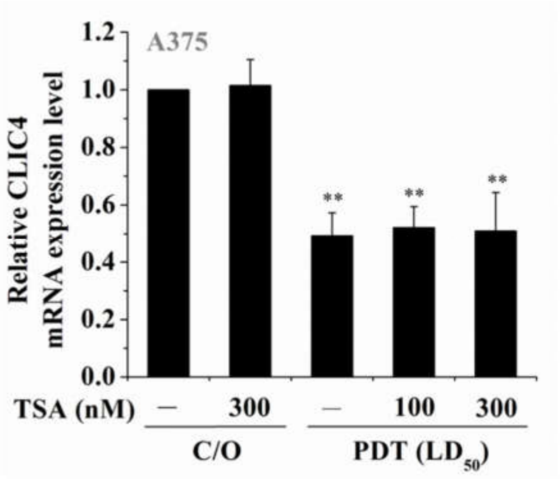

Figure S2. Inhibition of histone deacetylase (HDAC) activity by Trichostatin A (TSA) cannot suppress the PDT-induced reduction of *CLIC4*. A375 cells were pre-incubated with TSA for 2 hours prior to light irradiation. After ALA-PDT, total RNA samples were isolated from PDT-treated cells at 24 hours as indicated with or without pre-incubation of 100 and 300 nM TSA. The relative mRNA expression level of each gene was measured by RT-PCR and normalized to *β-actin*. The control group was cells only treated with ALA w/o light irradiation. The same volume of each PCR product was loaded, and band blots for each gene were cropped from different gels. Data represent from three independent experiments. Each bar shown is the mean fold change relative to control ± SD. Results are considered to be statistically significant at $^{**}p < 0.01$.

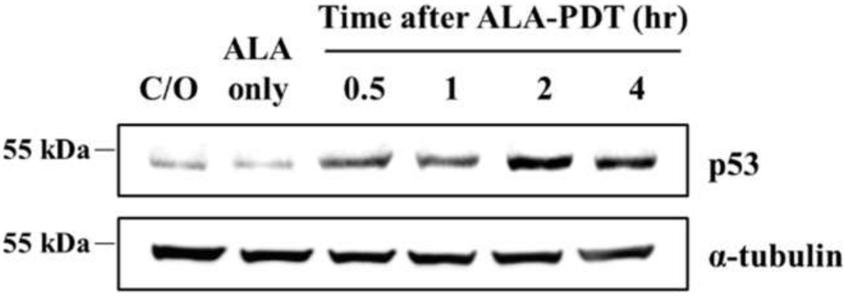

Figure S3. p53 activation following PDT in A375 cells. Cells were pre-incubated with 1 mM ALA for 3 hours and then exposed to specific wavelength (635±5 nm) of light under the dose of 2 J/cm^2. The cell lysates were collected from PDT-treated cells at the time indicated (0.5, 1, 2 and 4 hours after ALA-PDT). The protein expression level of p53 was analyzed by Western blotting, and α-tubulin was used as an internal control. For immunoblots, equal volume of each sample with the same concentration was loaded into each well, and the blots were cropped from different gels due to the target proteins have similar molecular weights.

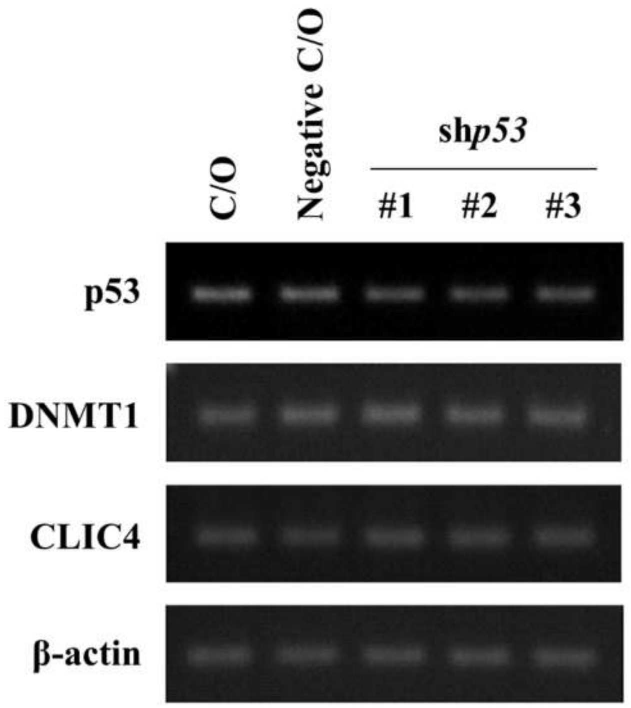

Figure S4. *DNMT1* and *CLIC4* mRNA expression levels in p53-knockdown A375 cells. Cells were transfected with either the sh*p53* (#1, #2, #3) or an empty shRNA vector (pLKO.1, as negative control). Total mRNA samples were isolated from treated cells. The relative mRNA expression level of each gene was measured by RT-PCR and normalized to *β-actin*. The same volume of each PCR product was loaded, and band blots for each gene were cropped from different gels.

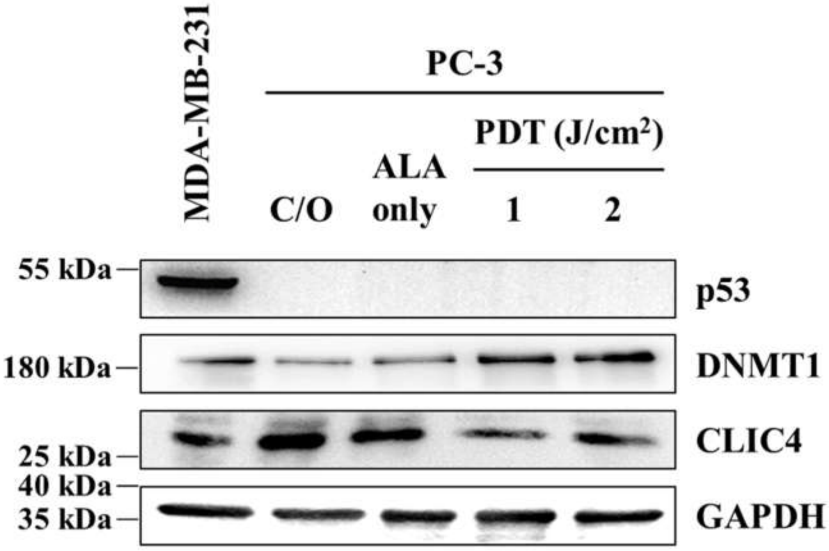

Figure S5. PDT induced DNMT1 expression and further suppressed CLIC4 expression in p53-null PC3 prostate cancer cells. Cells were pre-incubated with 1 mM ALA for 3 hours and then exposed to specific wavelength (635±5 nm) of light under the doses of 2 J/cm^2 and 4 J/cm^2. The cell lysates were collected from PDT-treated cells 24 hours after ALA-PDT. The protein expression levels of p53, DNMT1 and CLIC4 were analyzed by Western blotting, and GAPDH was used as an internal control. For immunoblots, equal volume of each sample with the same concentration was loaded into each well, and the blots were cropped from different sections of the same gel.

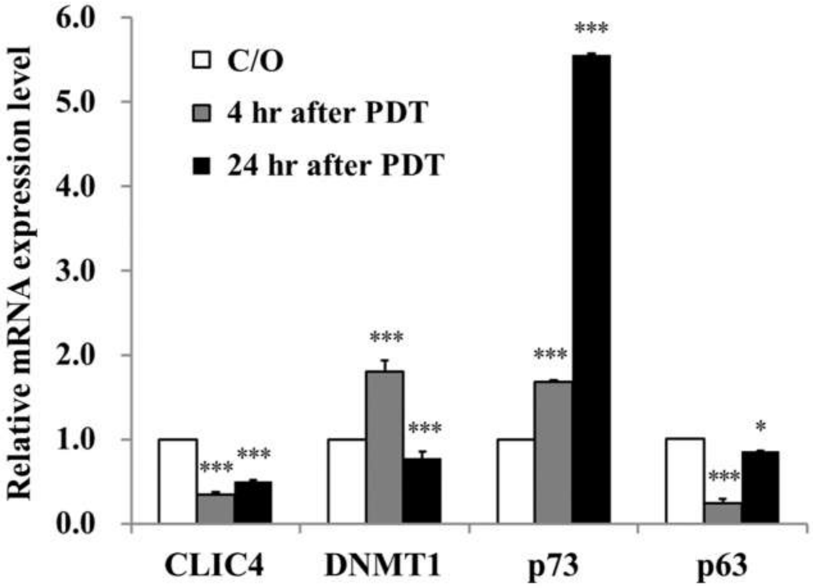

Figure S6. PDT increased *DNMT1* and *p73* expression in p53-null PC3 prostate cancer cells. Cells were pre-incubated with 1 mM ALA for 3 hours and then exposed to specific wavelength (635±5 nm) of light under the dose of 2 J/cm^2. Total RNA samples were isolated from PDT-treated cells at the time indicated (4 and 24 hours after ALA-PDT). The relative mRNA expression level of each gene was measured by real-time PCR and normalized to *GAPDH*. The control group was cells only treated with ALA w/o light irradiation. Data represent from three independent experiments. Each bar shown is the mean fold change relative to control ± SD. Results are considered to be statistically significant at *$p < 0.05$ and ***$p < 0.001$. Primer sequences used are as follows: CLIC4, 5'-gCAgTgATggTgAAAgCATAg-3' (forward) and 5'-TATAAATggTgggTgggTCC-3' (reverse); DNMT1, 5'-ACCgCTTCTACTTCCTCgAggCCTA-3' (forward) and 5'-gTTgCAgTCCTCTgTgAACACTgTgg-3' (reverse); p73, 5'-ACTTCAACgAAggACAgTCTgCT-3' (forward) and 5'-AATTCCgTCCCCACCTgTg-3' (reverse); p63, 5'-gTCCCAgAgCACACAgACAA-3' (forward) and 5'-gAggAgCCgTTCTgAATCTg-3' (reverse); GAPDH, 5'-gACCACAgTCCATgCCATCA-3' (forward) and 5'-gTCCACCACCCTgTTgCTgTA-3' (reverse).

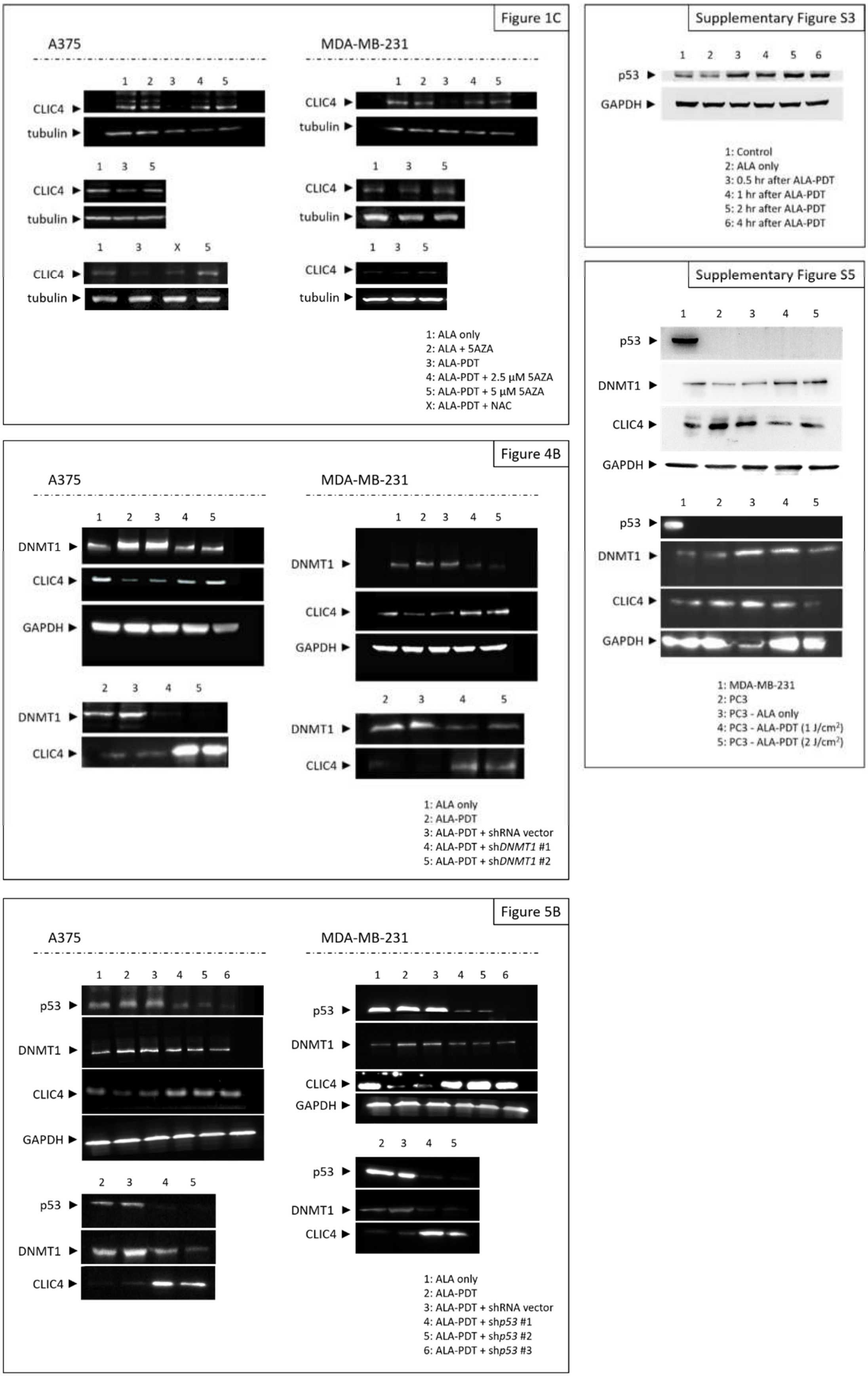

Figure S7. Original scans of immuno-blots used in main text and supplementary figures.

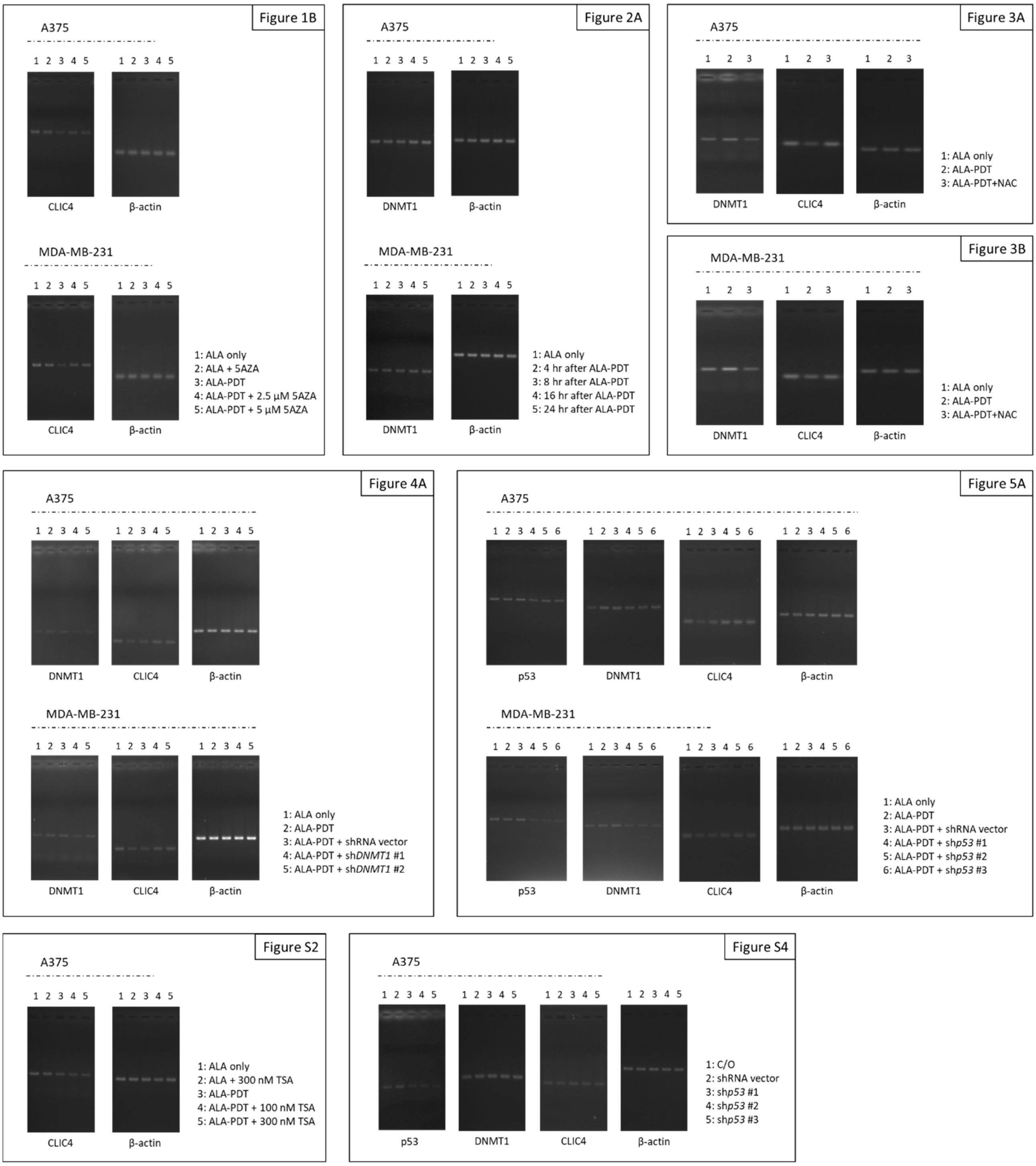

Figure S8. Original scans of electrophoresis gel used in main text and supplementary figures.
